# Fe_3_O_4_@Glycerol-Cu as a novel heterogeneous magnetic nanocatalyst for the green synthesis of 2-amino-4*H*-chromenes

**DOI:** 10.1038/s41598-022-26769-9

**Published:** 2022-12-22

**Authors:** Ahmad Poursattar Marjani, Fatemeh Asadzadeh, Aria Danandeh Asl

**Affiliations:** Department of Organic Chemistry, Faculty of Chemistry, Urmia University, Urmia, Iran

**Keywords:** Catalysis, Green chemistry

## Abstract

In the present study, the Fe_3_O_4_@Glycerol-Cu complex supported magnetically as a nanoparticle was prepared by grafting. Firstly, Fe_3_O_4_ NPs were synthesized by FeCl_3_.6H_2_O and FeCl_2_.4H_2_O according to the reported method, and subsequently, the prepared MNP with 3-chloropropyltrimethoxysilane. After that, the support-glycerol was functionalized on the surface of MNP-(CH_2_)_3_Cl for graft and stabilization of copper metal. Our purpose is to use the Fe_3_O_4_@Glycerol-Cu as a green, recoverable, novel, and affordable nanocatalyst in the effective synthesis of 2-amino-4*H*-chromenes. FT-IR, XRD, TGA, BET, VSM, TEM, and SEM–EDX techniques were examined to characterize this nanocatalyst. This result demonstrates that copper and organic compounds have appropriately reacted, with the support of MNP-(CH_2_)_3_Cl, and the crystalline structure have preserved in the MNP-(CH_2_)_3_Cl/Glycerol-Cu nanocatalyst confirmed the formation of the base Cu complex grafted on the surface of the nanoparticles. Finally, as can be seen, the nanoparticle size is 5–15 nm. This heterogeneous nanocatalyst illustrated excellent recyclable behavior, and can be used several times without notable reduction of its activity.

## Introduction

The researchers have considerably worried about the environmentally safe and health Issues of manufacturing from processes that include catalysts (catalytic processes). They have made significant endeavors to expand of environmentally pleasant recyclable catalysts for natural and organic variation, including multi-component reactions in the industrial and combinatorial fields^1–4^. In addition, the stabilization of diverse homogeneous catalysts onto different nanoparticles has appeared as a promising approach for amending stability and catalytic performance^5^. In recent decades, the stabilizing of diverse homogeneous catalysts onto different nanoparticles has transpired as a hopeful method to improve stability and catalytic performance^6^. The last few years have evidenced a significant advance in the expansion of different magnetic nanoparticles (MNPs) as effective and stable supports for catalysts in widely used fields owing to their interesting chemical and physical characteristics, including easy segregating of magnetic, large surface to volume ratio, and high performance of recyclability^7–11^. The application of diverse and combined magnetic metal oxide nanoparticles such as Fe_2_O_3_, Fe_3_O_4_, Al_2_O_3_, etc., has fascinated extensive regard in organic reactions as catalysts support^12^. Fe_3_O_4_ nanoparticles have appeared as excellent support for the stabilizing of different ligands and functional groups due to the attendance of high superficial density OH groups, high stability, easy magnetic separation, and biocompatibility^13–18^.

The recent rise in the popularity of biodiesel has significantly increased the demand for glycerol, an important oleochemical commodity. Glycerol forms complexes with many metal ions. It has been reported that copper ions through a reaction hydroxyl group glycerol (copper-glycerol complex) can be an intermediate product for certain chemical reactions^19–21^.

Amongst the heterocyclic scaffolds, chromenes are essential compounds of pharmacological and pharmacological significance that carried out anti-HIV, diuretic, antimalarial, and anticancer virtues^22–25^. Some of the chromenes have been extensively utilized as remedial and therapeutically beneficial factors, including acenocoumarol which functions as an anticoagulant^26^. As well, a wide diversity of chromenes are natural products found in many secondary metabolites such as anthocyanin, pigments, and flavonoids^27,28^. Also, a wide diversity of chromenes has been found in extensive applications such as cosmetics, pigments, and biocompatible agrochemicals^29^.

In line with our ongoing attempts in the field of catalysts^30–34^, in the present research, our purpose is the novel nanocatalyst production in the effective preparation of 2-amino-4*H*-chromenes. Fe_3_O_4_@Glycerol-Cu as a green nanocatalyst was employed for the first time with the effective formation of 2-amino-4*H*-chromenes through a one-pot, three-component reaction of malononitrile, cyclic 1,3-dicarbonyl compounds, and arylglyoxals. According to the results obtained, offered a plan for the preparation of 2-amino-4*H*-chromenes utilizing Fe_3_O_4_@Glycerol-Cu leads to favorable compounds with excellent efficiency and low reaction time, as well as, this green nanocatalyst under mild reaction demonstrated recyclable demeanor 6 times with the minor loss of its activity. According to our information, we presented an interesting magnetic nanocatalyst Fe_3_O_4_@Glycerol-Cu, a novel magnetic reusable nanocatalyst in the obtaining of 2-amino-4*H*-chromenes.

## Experimental section

### Materials and methods

All precursors and solvents for the synthesis were bought from Sigma-Aldrich and Merck and utilized without excess purification. FT-IR spectra were done using KBr pellets on the Nexus 670 apparatus. Nanostructure patterns were characterized by XRD measurement with a wavelength of 1.54 Å while these patterns were recorded in the range 10–80. The BET technique used nitrogen as the adsorption gas to measure the specific surface area. The properties measurement of the magnetic catalyst was performed by VSM. The morphology images of the nanocatalyst were recorded through a field-emission scanning electron microscope (FE-SEM). Elemental compositions of the nanocatalysts were affirmed using energy-dispersive X-ray (EDX) analysis.

### Synthesis Fe_3_O_4_ nanoparticles

One of the best methods for separating nanomaterials from a solution is magnetizing them. Magnetic nanoparticles have been synthesized with the following reaction:

Various solutions of FeCl_2_.4H_2_O (ferric chloride tetrahydrate) and FeCl_3_.6H_2_O (ferrous chloride hexahydrate) were provided with a molar ratio of 1 to 2. In this, the usual method, FeCl_2_.4H_2_O (1.5 g) and FeCl_3_.6H_2_O (3 g), were mixed in deionized water (50 mL). Next, NH_4_OH (ammonium hydroxide, 10 mL of 25% solution) was added dropwise to the above solution and stirred vigorously (50 min, 80 °C, and 700 rpm). A black precipitate is the result of the reaction. The obtained nanoparticles were washed with EtOH and eventually dried (under vacuum conditions at 60 °C for 24 h).

### Preparation of MNP-(CH_2_)_3_Cl

The prepared MNP (1.6 g) with 3-chloropropyltrimethoxysilane (1.7 g) was added to ethanol (35 mL), and the compound was stirred under reflux conditions at 70 °C for 24 h. Afterward, the obtained solid was washed with ethanol, and eventually, the solid was dried (under vacuum) to acquire MNP–(CH_2_)_3_–Cl.

### Preparation of support-glycerol

The prepared MNP-(CH_2_)_3_Cl (1.2 g) in a 50 mL round-bottom flask with glycerol (25 mL) and EtOH (40 mL) were refluxed at 70 °C for 24 h. Then, the obtained product was separated by the magnet and frequently washed with EtOH. Eventually, the product was dried at 70 °C for 20 h in an oven.

### Synthesis of Fe_3_O_4_@Glycerol-Cu

The made support-glycerol (1.5 g) was combined with CuSO_4_.5H_2_O (0.7 g) in EtOH (40 mL) and reflux conditions at 70 °C for 24 h. As soon as the reaction was completed, the Fe_3_O_4_@Glycerol-Cu nanoparticles were collected by an external magnet. Finally, the separated product was washed frequently with EtOH and DW to eliminate the unwanted excess materials and then dried at 70 °C for 15 h.

### Preparation of 2-amino-4*H*-chromenes

First, a combination of arylglyoxals (1 mmol), malononitrile (1.1 mmol), cyclic 1,3-dicarbonyl compounds (1 mmol), and magnetic nanocatalyst (10 mg) were added to a flask containing ethanol (10 mL) and the reaction contents were stirred at room temperature (Table 2, appropriate reaction time in the range of 15–20 min) and then the reaction progression was evaluated using thin-layer chromatography. After completing the reaction, the nanocatalyst was segregated by a magnet and washed with acetone or water until the following reaction. Finally, ^1^H-NMR spectroscopy and FT-IR analysis were used to identify the products, and the precipitate was filtered and recrystallized from ethanol to get the desired 2-amino-4*H*-chromenes in high yields.

## Results and discussions

**Figure 1:**
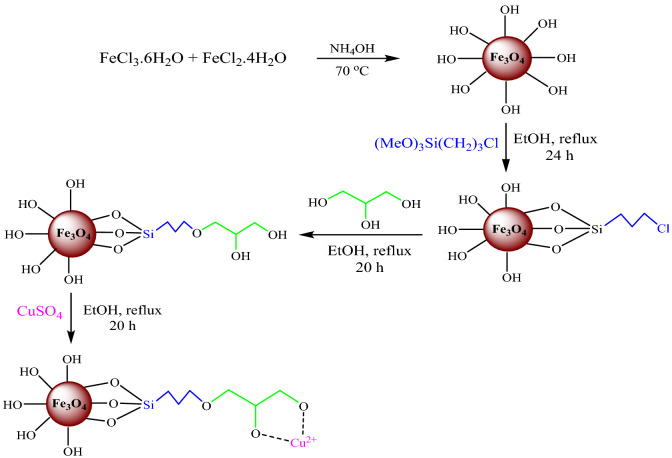
Schematic stepwise preparation of Fe_3_O_4_@Glycerol-Cu magnetic nanocatalyst.

Our study on the application of novel retrievable and heterogeneous magnetic nanocatalysts in organic transformations encouraged us to report a new heterogeneous catalyst (Fe_3_O_4_@Glycerol-Cu) and to study its utilization in the preparation of 2-amino-4*H*-chromenes. Figure 1, represents a concise route of the magnetic catalyst preparation process.

### Catalyst characterization

The newly prepared nanocatalyst structure was characterized using various techniques, including FT-IR (Fourier transform infrared), TGA (Thermogravimetric analysis), XRD (X-ray diffraction), BET (Brunauer–Emmett–Teller), VSM (vibrating-sample magnetometer), EDX (energy dispersive X-ray spectroscopy) and SEM (scanning electron microscopy).

#### FT-IR analysis

**Figure 2:**
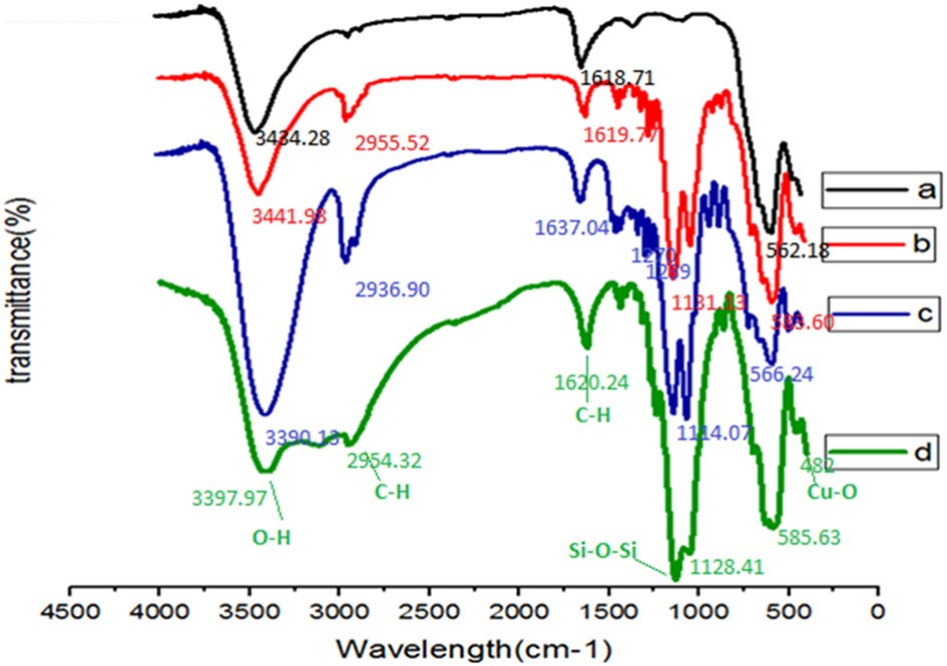
FT-IR spectra of Fe_3_O_4_ (curve **a**), MNP–(CH_2_)_3_Cl (curve **b**), MNP–(CH_2_)_3_Cl/Glycerol (curve **c**) and MNP–(CH_2_)_3_Cl/Glycerol-Cu (curve **d**).

Figure 2, illustrates the FT-IR spectra acquired for Fe_3_O_4_ (curve a), MNP–(CH_2_)_3_Cl (curve b), MNP–(CH_2_)_3_Cl/Glycerol (curve c), and MNP–(CH_2_)_3_Cl/Glycerol-Cu (curve d). Curve a, shows the FT-IR spectrum of Fe_3_O_4_, and peaks that appeared at 562, 1618, and 3436 cm^−1^ are attributed to Fe–O vibrations, O–H bending, and O–H stretching, respectively. Curve b, represents the characteristic absorption bands at 2955, 800–1200 cm^−1^ correspond to aliphatic C–H stretching, Si–O stretching, and Si–O–Si symmetric and asymmetric stretching, respectively. The peaks in the region of 3390, 1239 and 1270 cm^−1^ correspond to vibrations of O–H stretching vibrations (Change the peak size compared to a and b) and C–O stretching vibrations relating to glycerol (curve c). Moreover, the peak observed at 482 cm^−1^ in the spectrum (curve d) can be assigned to Cu–O vibration. The advent of the characteristic absorption bands in all the curves a–d, illustrates the successful immobilization of Cu and the organic moieties on the surface of Fe_3_O_4_ nanoparticles.

#### XRD analysis

**Figure 3:**
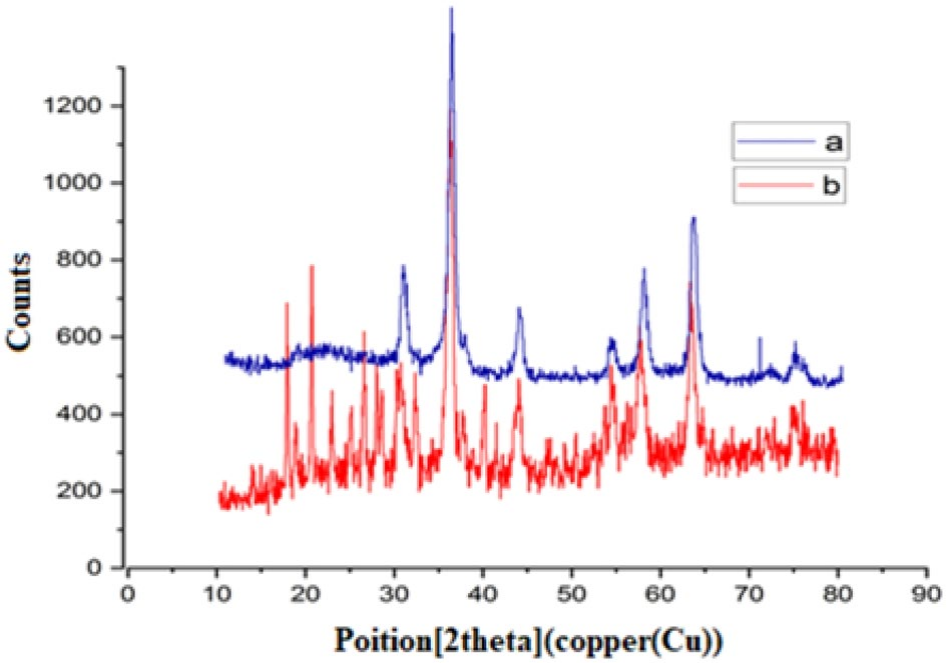
The XRD pattern of MNP–(CH_2_)_3_Cl (**a**) and MNP–(CH_2_)_3_Cl/Glycerol-Cu (**b**).

The nanoparticle's size and crystalline nature of MNP-(CH_2_)_3_Cl/Glycerol-Cu were characterized by XRD. Figure 3a, exhibits the XRD pattern of MNP–(CH_2_)_3_Cl that Bragg's peaks were observed at 2θ = 31, 36, 43.5, 53.9, 57.5 and 63, that these angles correspond to the (220), (311), (400), (422), (511) and (440) crystal planes, respectively. These peaks indicate the presence of Fe_3_O_4_ in the compound. Figure 3b shows the XRD pattern of MNP–(CH_2_)_3_Cl/Glycerol-Cu that Bragg's peaks were observed at 2θ = 18, 21, 43.5, 53.9, 57.5 and 63. Also, the peak intensity at common angles (Fig. 3a,b) has been reduced. This result demonstrates that copper and organic compounds have adequately reacted, with the support (MNP–(CH_2_)_3_Cl), and the crystalline structure has preserved in the MNP–(CH_2_)_3_Cl/Glycerol-Cu nanocatalyst. Also, the average crystallite size of nanoparticles by utilizing the Debye–Scherer equation which is shown in Eq. (1), can be analyzed by the XRD pattern.

1 $$D = \frac{K\lambda }{\beta cos\theta }$$

D in the mentioned equation represents the average crystalline, and K shows a dimensionless shape factor of about 0.9, λ is the X-ray wavelength; β is the line broadening at half the maximum intensity, as well as θ, is the Bragg angle^35^.

#### TGA analysis

**Figure 4:**
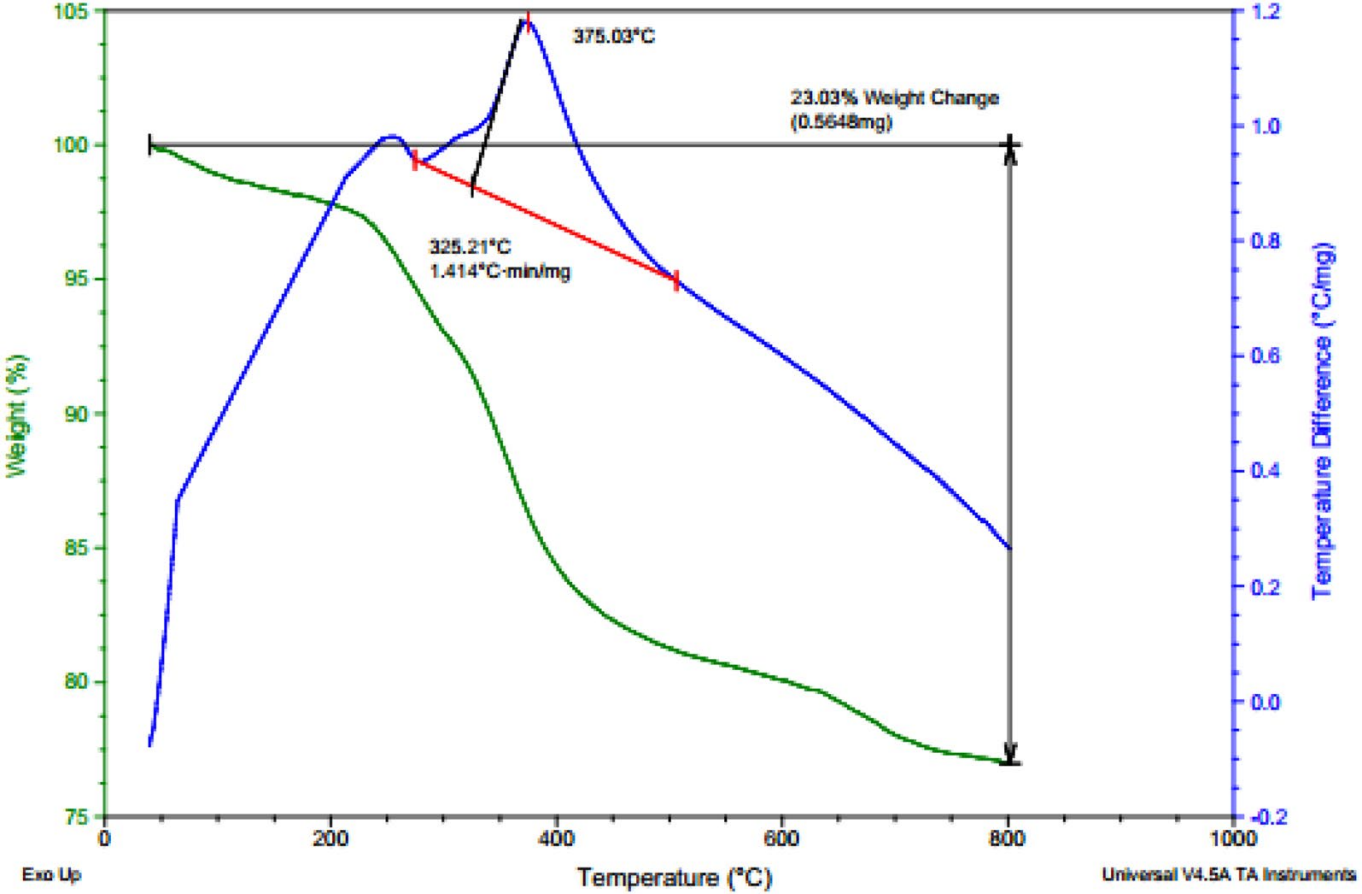
Thermal gravimetric analysis of the Fe_3_O_4_@Glycerol-Cu nanocatalyst.

TGA (Thermal gravimetric analysis) was utilized on the Fe_3_O_4_@Glycerol-Cu nanocatalyst to study its thermal stability. According to Fig. 4, the first weight loss happens below 200 °C because of the removal of the structure O–H and remaining organic solvents. The next weight loss in the range of 150–450 °C is likely attributed to the removal of the Cu complex and the Fe_3_O_4_-grafted organic materials. In the final thermal step beyond 450 °C the change of the crystal phase and complete dissociation of the catalyst occurs.

#### N_2_-physical adsorption studies

**Figure 5:**
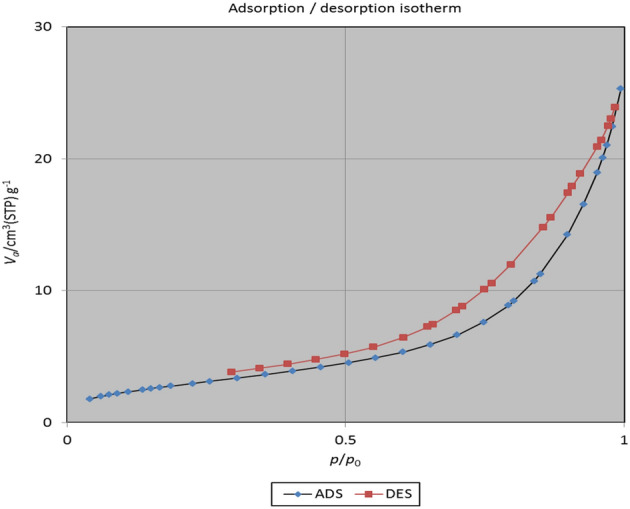
Nitrogen adsorption/desorption isotherms of the Fe_3_O_4_@Glycerol-Cu magnetic nanocatalyst.

Figure 5, illustrates the nitrogen adsorption/desorption isotherms of the Fe_3_O_4_@Glycerol-Cu magnetic nanocatalyst. The hole property was evaluated by nitrogen adsorption–desorption. The particular surface area was computed using the BET for the synthesized Fe_3_O_4_@Glycerol-Cu nanocatalyst, and its value was 10.785 m^2^g^−1^. The hole volume is 2.4779 cm^3^g^−1^. The related hole size spread of the magnetic nanocatalyst was determined as 14.119 nm, using the BJH technique. These results indicate that the Fe_3_O_4_@Glycerol-Cu magnetic nanocatalyst was acquired as a mesoporous type (2 < D_v_ < 50 nm, D_v_ is the particle diameter of the volume distribution).

#### VSM analysis

**Figure 6:**
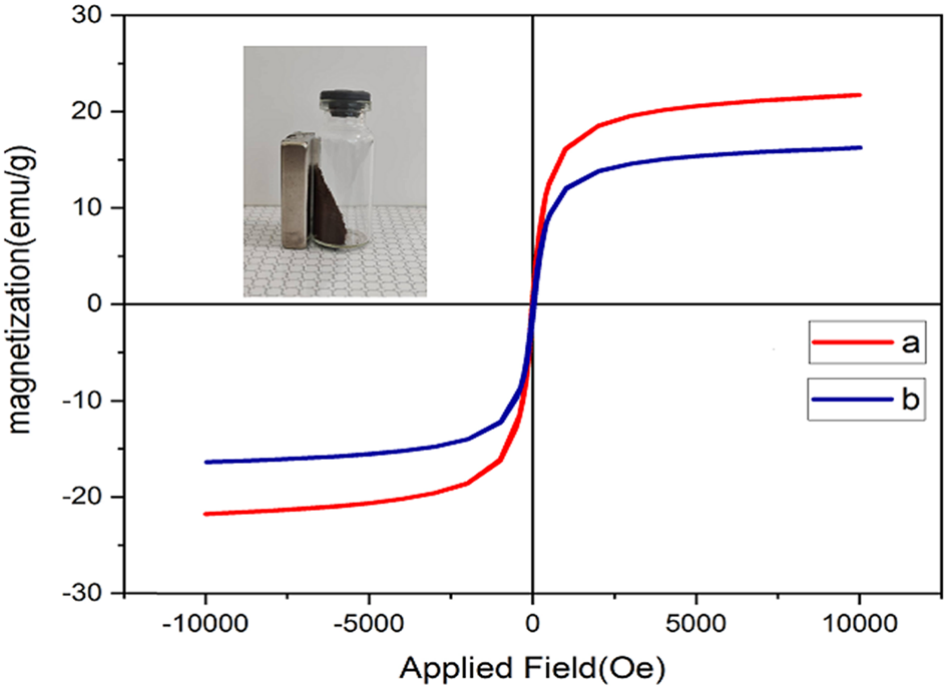
The VSM data for MNP–(CH_2_)_3_Cl (curves **a**) and Fe_3_O_4_@Glycerol-Cu (curves **b**).

Figure 6, shows the magnetization behavior and Feature of Fe_3_O_4_@Glycerol-Cu, with a vibrating sample magnetometer (VSM) at room temperature. Also, Fig. 6, illustrates the VSM data obtained for MNP–(CH_2_)_3_Cl and Fe_3_O_4_@Glycerol-Cu in curves a and b, respectively. The specific saturation magnetizations of MNP–(CH_2_)_3_Cl and Fe_3_O_4_@Glycerol-Cu are measured to be 30 and 20 emu/g^−1^ respectively. The Negligible reduction (10 emu/g^−1^) observed in Ms of the Fe_3_O_4_@Glycerol-Cu nanoparticles in comparison to the Ms value of the bare MNP–(CH_2_)_3_Cl nanoparticles was most likely related to the presence of the coated materials on the surface of the Fe_3_O_4_ nanoparticles.

#### EDX analysis

**Figure 7:**
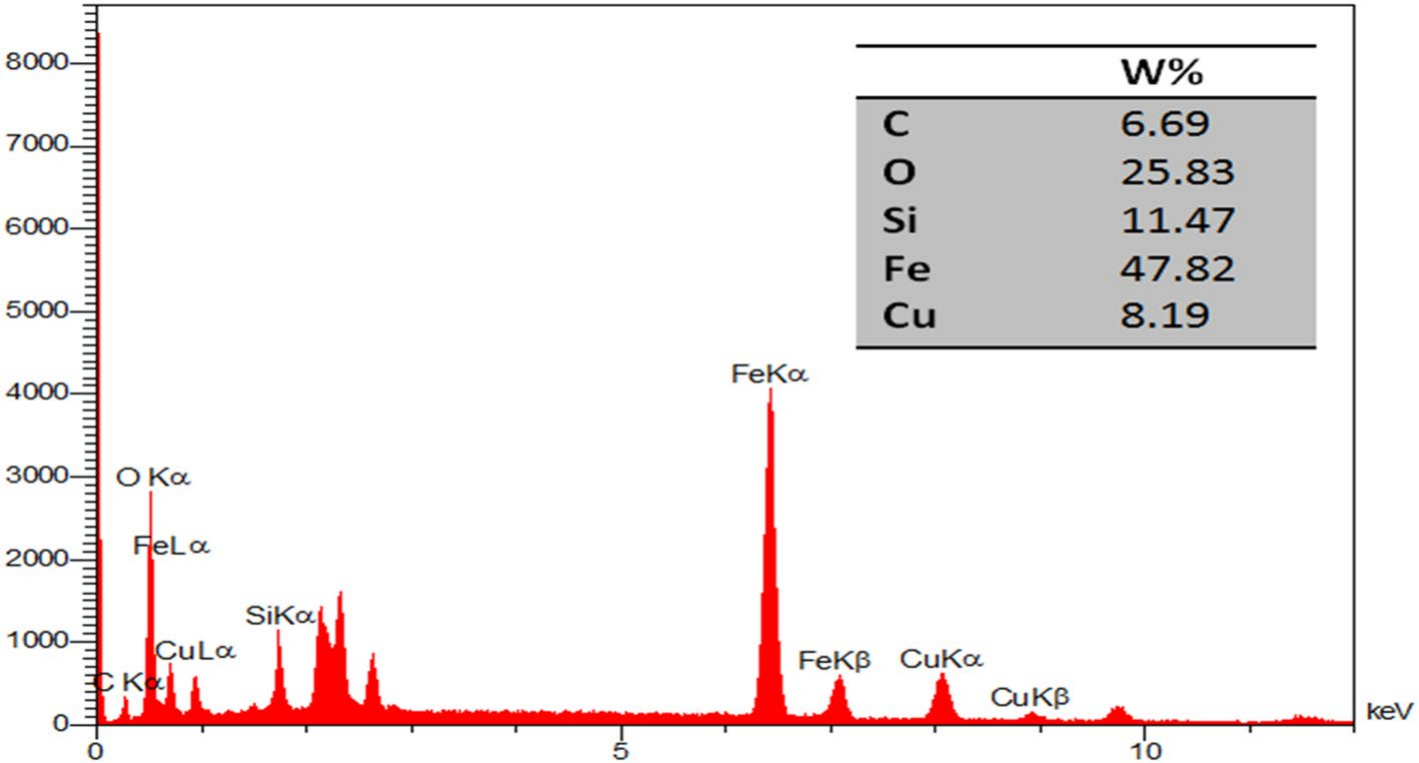
EDX results of the Fe_3_O_4_@Glycerol-Cu nanocatalyst.

The EDX data illustrate the presence of Fe, Si, C, O, and Cu in Fe_3_O_4_@Glycerol-Cu (Fig. 7). As is evident from the spectrum, the existence of Cu, Fe, O, C, and Si elements with wt% of 8.19, 47.82, 25.83, 6.69 and 11.47 were detected in MNP-(CH_2_)_3_Cl/Glycerol-Cu, respectively. The advent of the peaks related to the O and Cu atoms confirmed the formation of the base Cu complex grafted on the surface of the nanoparticles.

#### SEM analysis

**Figure 8:**
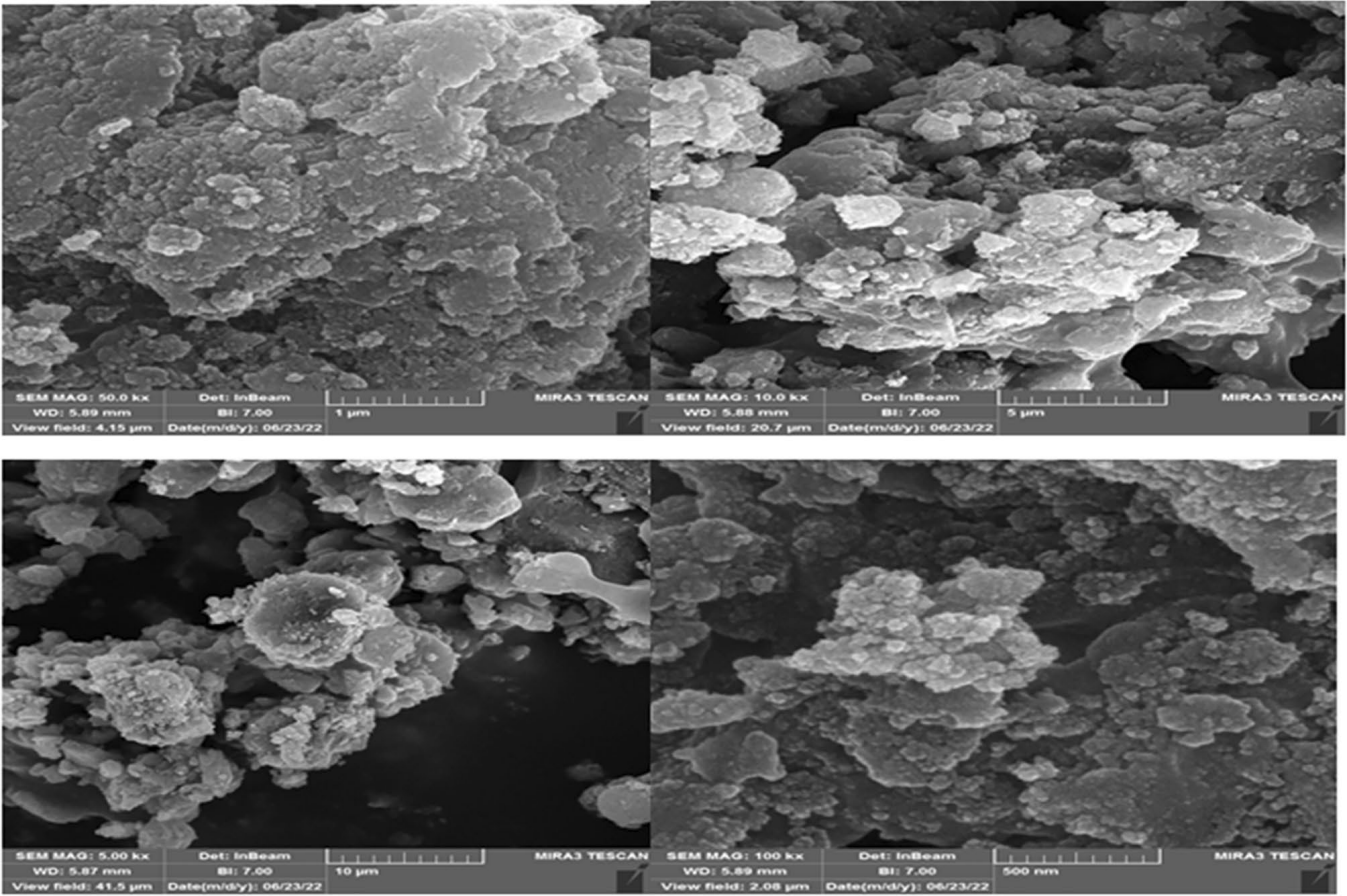
SEM image of synthesized Fe_3_O_4_@Glycerol-Cu.

Figure 8, shows the morphology and particle size of Fe_3_O_4_@Glycerol-Cu using SEM (scanning electron microscopy). These images indicate the spherical structure and demonstrate that the nanocatalyst was made of uniform nanometer particles. Finally, as can be seen, the nanoparticle size is 18 nm.

#### TEM analysis

**Figure 9:**
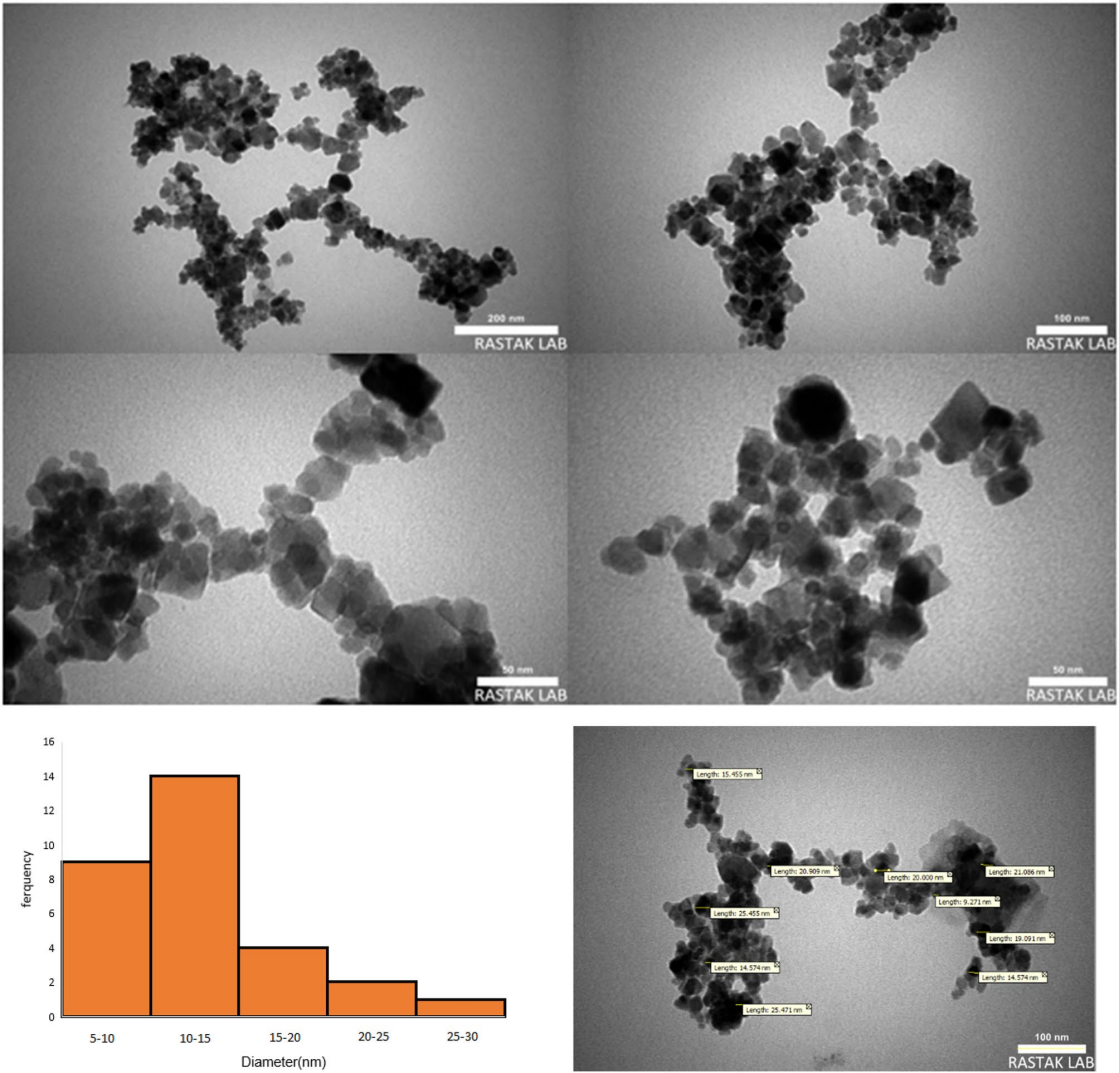
TEM image of Fe_3_O_4_@Glycerol-Cu.

Transmission electron microscopy (TEM) has shown to be a useful tool for analyzing the size and shape distribution of particles. TEM image of Fe_3_O_4_@Glycerol-Cu exhibits that the diameter size of the as-prepared sample is in the range of 5–15 nm (Fig. 9). Furthermore, distinct structures in which the Fe_3_O_4_ spherical cores are visible as dark regions coated by bright outer shells can be detected, also, the distances show the cross links that caused the holes to appear.

### Synthesis of 2-amino-4***H***-chromenes by amounts of Fe_3_O_4_@Glycerol-Cu

After obtaining and evaluating the magnetic nanocatalyst structure, it was investigated that the catalytic activity of Fe_3_O_4_@Glycerol-Cu as an effective catalyst in the direct formation of 2-amino-4*H*-chromenes. Thus, to find out a simple and biocompatible method for the synthesis of target products in the presence of Fe_3_O_4_@Glycerol-Cu as a recyclable and stable nanocatalyst in the reaction of 4-fluoroarylglyoxal (**1b**), malononitrile (**2**), and cyclohexane-1,3-dione (**3a**) with molar ratio: 1:1.1:1 was explored to create optimal conditions. Optimization of the nanocatalyst in the synthesis of product **4b,** using different catalytic amounts of Fe_3_O_4_@Glycerol-Cu, is demonstrated in Table 1. Outstanding results were obtained with 4-fluoroarylglyoxal (1 mmol), malononitrile (1.1 mmol), cyclohexane-1,3-dione (1 mmol) in the presence of Fe_3_O_4_@Glycerol-Cu (10 mg) and ethanol (5 mL) at room temperature (Table 1, entry 3). The use of lower and higher amounts of nanocatalyst (5 and 15 mg) had not affected the result (Table 1, entries 2 and 4, respectively). The optimal time for the reaction is 15 min. TLC results confirmed that no impurities were observed in the reaction.

**Table 1:** Effects of various parameters on the synthesis of **4b**.


Entry	Catalyst (mg)	Solvent	Temperature (°C)	Time (min)	Yield (%)
1	0	Solvent-free	60	120	Trace
2	5	EtOH	RT	67	87
3	**10**	**EtOH**	**RT**	**16**	**95**
4	15	EtOH	RT	15	95
5	10	H_2_O	70	75	40
6	10	CH_3_CN	60	75	45

Reaction conditions: Arylglyoxals (1 mmol), malononitrile (1.1 mmol), and cyclic 1,3-dicarbonyl compounds (1 mmol) with various solvents.

Significant values are in bold.

The reaction was carried out with 95% yield. As indicated in Table 2, the suggested method is generalizable and includes different functional groups.

**Table 2:** Fe_3_O_4_@Glycerol-Cu nanocatalyst the one-pot synthesis of products **4a–h** in EtOH.


Entry	X	R	Product	Time (min)	Yield (%)	M.p. (°C)	
						Obsd	References
1	4-Cl	H	**4a**	16	94	173–175	176–178^36^
2	4-F	H	**4b**	16	95	170–172	169–171^36^
3	4-Me	H	**4c**	15	91	212–214	216–218^36^
4	4-MeO	H	**4d**	19	90	208–210	210–212^36^
5	4-Cl	Me	**4e**	15	94	178–180	181–183^36^
6	4-F	Me	**4f**	15	96	182–184	184–186^36^
7	4-Me	Me	**4g**	15	98	173–175	177–179^36^
8	4-MeO	Me	**4h**	20	91	140–142	141–143^36^

**Figure 10:**
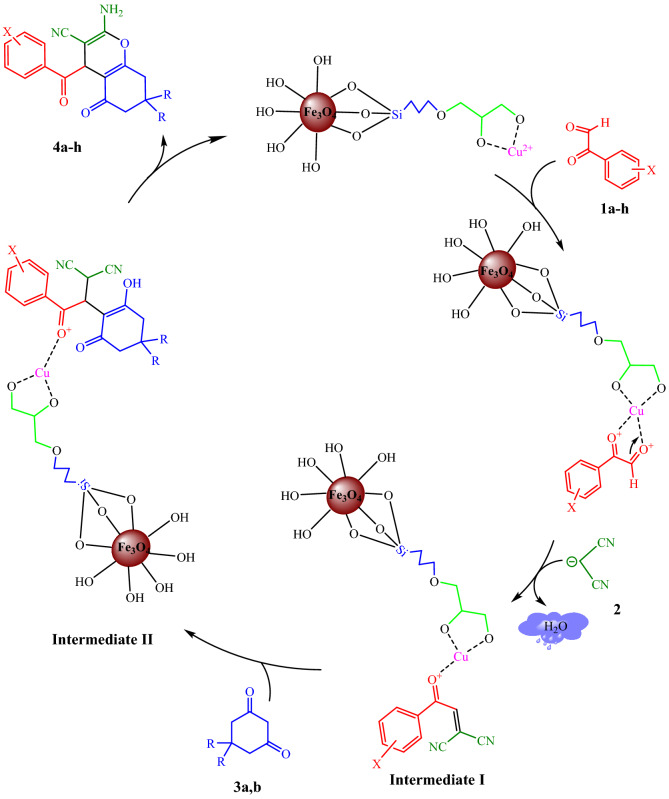
The possible mechanism for the obtaining of compounds **4a–h**.

As shown in Fig. 10, an appropriate pathway for forming of compounds **4a–h** in the presence of prepared Fe_3_O_4_@Glycerol-Cu nanocatalyst is proposed. Initially, the oxygen atom in carbonyl groups of arylgloxals **1a–h** coordinated with Cu in the head of the nanocatalyst. Then *Knoevenagel* condensation of the activated formyl group of arylglyoxals **1a-h**, with malononitrile (**2**), leads to form the intermediate **I** by the removal of the water molecule**.** After that, intermediate **II** is created via the *Michael* addition of cyclic 1,3-dicarbonyl compounds **3a,b** with intermediate **I**. Subsequently, heterocyclization occurred and the corresponding products **4a-h** were produced.

The efficiency and capability of the Fe_3_O_4_@Glycerol-Cu system in synthesizing of chromenes were compared with the obtained result from other data (Table 3). As illustrated, Fe_3_O_4_@Glycerol-Cu is the best nanocatalyst in synthesizing compounds **4a–h** in a green solvent under moderate conditions. This nanocatalyst has significant features such as biocompatibility, selectivity, low cost, and chemically stable materials.

**Table 3:** The catalytic performance comparison of Fe_3_O_4_@Glycerol-Cu with past research.

Entry	Catalyst	Solvent	Time (min)	Yield (%)	References
1	L-proline	EtOH	95	92	^36^
2	NaOH	EtOH	240	66	^37^
3	(SB-DBU)Cl	EtOH	25	95	^38^
4	Piperidine	EtOH	120	80	^39^
5	DMAP	EtOH	15	94	^40^
6	SB-DABCO	EtOH	30	93	^41^
7	CuSO_4_.5H_2_O	H_2_O	60	95	^42^
8	GO/α-Fe_2_O_3_/CuL	Solvent Free	60	98	^43^
9	**Fe**_**3**_**O**_**4**_**@Glycerol-Cu**	**EtOH**	**15**	**95**	**This research**

Significant values are in bold.

### Recyclability of the nanocatalyst

**Figure 11:**
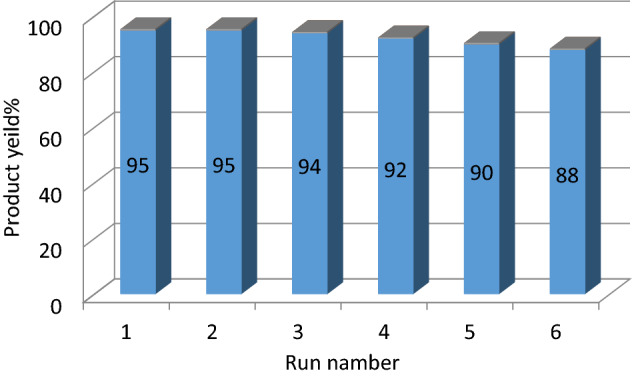
Recycling of Fe_3_O_4_@Glycerol-Cu in the construction of 2-amino-4*H*-chromene.

The reuse of nanocatalysts is a significant benefit to industrial applications. We checked out the reusability of the Fe_3_O_4_@Glycerol-Cu nanocatalyst for the reaction between arylglyoxals, malononitrile, and cyclic 1,3-diketones under optimal reaction conditions. The separated nanocatalyst (by a magnet) was washed with EtOH several times and dried at 70 °C. Finally, the produced nanocatalyst was used six consecutive times without loss of reactivity (Fig. 11).

Considering the similarity of the FT-IR spectra obtained from the catalyst of the first stage and the 6^th^ stage of catalyst recycling, with the peaks of the spectrum of the newly synthesized catalyst, it can be concluded that the efficiency decreases in the last stages of recycling due to the change in the ligand structure and other related factors. It is not related to the reduction of efficiency, but it is related to the reduction of copper as a result of washing in the recycling cycle. The yield of the product obtained in the final cycle was approximately 88%, which indicates that the leaching of copper from the recovered catalyst was negligible.

### Hot filtration

**Figure 12:**
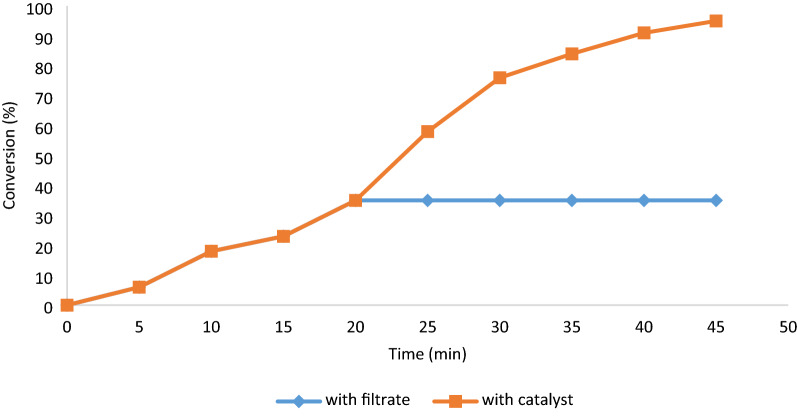
Hot filtration test for Fe_3_O_4_@Glycerol-Cu.

The hot filtration was carried out for the preparation of products **4a–h** with using of arylglyoxals, malononitrile, and cyclic 1,3-diketones. In the first twenty minutes of the reaction, the yield is 33%. Then, the nanocatalyst was isolated, and the filtrate was allowed to react more. In the end, no other reaction was observed (Fig. 12).

## Conclusion

Briefly, in the present study, the Fe_3_O_4_@Glycerol-Cu nanocatalyst was synthesized utilizing the grafting technique. The prepared product (Fe_3_O_4_@Glycerol-Cu) was structurally evaluated by XRD, FT-IR, TGA, BET, VSM, TEM, and SEM–EDX. The advent of the characteristic absorption bands in all the curves of FT-IR analysis illustrates the successful immobilization of Cu and the organic moieties on the surface of Fe_3_O_4_ nanoparticles. The EDX data illustrate related to the O, and Cu atoms confirmed the formation of the base Cu complex grafted on the surface of the nanoparticles. The catalytic activity of these nanoparticles prepared as Lewis acid heterogeneous catalysts for the preparation of 2-amino-4*H*-chromenes was investigated through one-pot green synthesis. Therefore, the suggested route for obtaining 2-amino-4*H*-chromenes using Fe_3_O_4_@Glycerol-Cu gives favorable products with higher yields and short reaction times. Finally, this green nanocatalyst is helpful for the synthesis of these kinds of compounds based on availability and selectivity (Supplementary information S1).

## Supplementary Information

Supplementary Information.

## Data Availability

All data generated or analyzed during this study are included in this published article.

## References

[1] Nasir Baig, R. B. & Varma, R. S. Copper on chitosan: a recyclable heterogeneous catalyst for azide-alkyne cycloaddition reactions in water. *Green Chem*. **15**, 1839–1843 (2013).

[2] Someshwar DD, Vedavati GP, Yeon TJ (2012). Supported copper triflate as an efficient catalytic system for the synthesis of highly functionalized 2-naphthol Mannich bases under solvent free condition. Tetrahedron Lett..

[3] Rambhau PG, Ambarsing PRA (2013). A review on recent progress in multicomponent reactions of pyrimidine synthesis. Drug Invention Today..

[4] Yunyun L, Rihui Z, Jie-Ping W (2013). Water-promoted synthesis of enaminones: Mechanism investigation and application in multicomponent reactions. Synth. Commun..

[5] Wu X, Lu C, Zhou Z, Yuan G, Xiong R, Zhang X (2014). Green synthesis and formation mechanism of cellulose nanocrystal-supported gold nanoparticles with enhanced catalytic performance. Environ. Sci. Nano..

[6] Sajjadifar, S., Rezayati, S., Arzehgar, Z., Abbaspour, S. & Torabi Jafroudi, M. Applications of iron and nickel immobilized on hydroxyapatite-core-shell γ-Fe_2_O_3_ as a nanomagnetic catalyst for the chemoselective oxidation of sulfides to sulfoxides under solvent-free conditions. *J. Chin. Chem. Soc.* **65**, 960–969 (2018).

[7] Taghavi Fardood, S., Ramazani, A., Moradnia, F., Afshari, Z., Ganjkhanlu, S. & Yekke Zare, F. Green synthesis of ZnO nanoparticles via sol-gel method and investigation of its application in solvent-free synthesis of 12-aryl-tetrahydrobenzo[*α*]xanthene-11-one derivatives under microwave irradiation. *Chem. Methodol.* **3**, 632–642 (2019).

[8] Dálaigh CÓ, Corr SA, Gun'ko Y, Connon SJ (2007). A Magnetic-nanoparticle-supported 4-*N, *N-dialkylaminopyridine catalyst: Excellent reactivity combined with facile catalyst recovery and recyclabilit*y*. Angew Chem. Int. Ed..

[9] Shi F, Tse MK, Pohl MM, Bruckner A, Zhang S, Beller M (2007). Tuning catalytic activity between homogeneous and heterogeneous catalysis: Improved activity and selectivity of free Nano-Fe_2_O_3_ in selective oxidations. Angew. Chem. Int. Ed..

[10] Clark JH, Macquarrie DJ (2002). Handbook of Green Chemistry and Technology.

[11] Zheng X, Luo S, Zhang L, Cheng J-P (2009). Magnetic nanoparticle supported ionic liquidcatalysts for CO_2_ cycloaddition reactions. Green Chem..

[12] Azarifar, A., Nejat-Yami, R., Al Kobaisi, M. & Azarifar, D. Magnetic La_0.7_Sr_0.3_MnO_3_ nanoparticles: Recyclable and efficient catalyst for ultrasound-accelarated synthesis of 4*H*-chromenes, and 4*H*-pyrano[2,3-*c*]pyrazoles. *J. Iran. Chem. Soc*. **10**, 439–446 (2013).

[13] Govan JE, Gun'ko YK (2014). Recent advances in the application of magnetic nanoparticles as a support for homogeneous catalysts. Nanomaterials.

[14] Chng LL, Erathodiyil N, Ying JY (2013). Nanostructured catalysts for organic transformations. Acc Chem. Res..

[15] Baig RN, Varma RS (2013). Magnetically retrievable catalysts for organic synthesis. Chem. Commun..

[16] Kooti M, Karimi M, Nasiri E (2018). A novel copper complex supported on magnetic reduced graphene oxide: an efficient and green nanocatalyst for the synthesis of 1-amidoalkyl-2-naphthol derivatives. J. Nanopart Res..

[17] Ghorbani-Choghamarani A, Tahmasbi B, Moradi Z (2017). S-Benzylisothiourea complex of palladium on magnetic nanoparticles: A highly efficient and reusable nanocatalyst for synthesis of polyhydroquinolines and Suzuki reaction. Appl. Organomet. Chem..

[18] Wang D, Astruc D (2014). Fast-growing field of magnetically recyclable nanocatalysts. Chem. Rev..

[19] Ashraf MA, Liu Z, Peng W-X, Zhou L (2020). Glycerol Cu(II) complex supported on Fe_3_O_4_ magnetic nanoparticles: A new and highly efficient reusable catalyst for the formation of aryl-sulfur and aryl-oxygen bonds. Catal. Lett..

[20] La Penna G, Machetti F, Proux O, Rossi G, Stellato F, Morante S (2021). Cu(II)–glycerol–*N*-ethylmorpholine complex stability revealed by X-ray spectroscopy. J. Phys. Chem. C..

[21] Yee CM, Hassan HA, Hassan ZAA, Ismail H (2012). Zinc glycerolate: potential active for topical application. J. Oil Palm. Res..

[22] Khaksar S, Rouhollahpour A, Talesh SM (2012). A facile and efficient synthesis of 2-amino-3-cyano-4*H*-chromenes and tetrahydrobenzo[*b*]pyrans using 2,2,2-trifluoroethanol as a metal-free and reusable medium. J. Fluorine Chem..

[23] Gao S, Tsai CH, Tseng C, Yao CF (2008). Fluoride ion catalyzed multicomponent reactions for efficient synthesis of 4*H*-chromene and *N*-arylquinoline derivatives in aqueous media. Tetrahedron.

[24] Kumar D, Sharad VB, Dube SU, Kapur S (2009). A facile one-pot green synthesis and antibacterial activity of 2-amino-4*H*-pyrans and 2-amino-5-oxo-5,6,7,8-tetrahydro-4*H*-chromenes. J. Eur. Med. Chem..

[25] Bolognese, A., Correale, G., Manfra, M., Lavecchia, A., Mazzoni, O., Novellino, E., La Colla, P., Sanna, G. & Loddo, R. Antitumor agents. 3. Design, synthesis, and biological evaluation of new pyridoisoquinolindione and dihydrothienoquinolindione derivatives with potent cytotoxic activity. *J. Med. Chem.* **47**, 849–858 (2004). 10.1021/jm030918b 14761187

[26] Joule JA, Mills K, Smith GF (1995). Heterocyclic Chemistry, 3rd ed.

[27] Morita N, Arisawa M (1976). Flavonoids: Chemistry and biochemistry. Heterocycles.

[28] Schmid H (1954). Natürlich vorkommende chromone. Chem. Org. Naturst..

[29] Peng Y, Song G (2007). Amino-functionalized ionic liquid as catalytically active solvent for microwave-assisted synthesis of 4*H*-pyrans. Catal. Commun..

[30] Kafi-Ahmadi L, Poursattar Marjani A, Nozad E (2021). Ultrasonic‐assisted preparation of Co_3_O_4_ and Eu‐doped Co_3_O_4_ nanocatalysts and their application for solvent‐free synthesis of 2‐amino‐4*H*‐benzochromenes under microwave irradiation. Appl. Organomet. Chem..

[31] Majidi Arlan, F., Poursattar Marjani, A., Javahershenas, R. & Khalafy, J. Recent developments in the synthesis of polysubstituted pyridines via multicomponent reactions using nanocatalysts. *New J. Chem.* **45**, 12328–12345 (2021)

[32] Azimi F, Poursattar Marjani A, Keshipour S (2021). Fe(II)-phthalocyanine supported on chitosan aerogel as a catalyst for oxidation of alcohols and alkyl arenes. Sci. Rep..

[33] Khashaei, M. Kafi-Ahmadi, L. Khademinia, S. Poursattar Marjani, A. & Nozad. E. A facile hydrothermal synthesis of high-efficient NiO nanocatalyst for preparation of 3,4-dihydropyrimidin-2(1*H*)-ones. *Sci. Rep.* **12**, 8585 (2022). 10.1038/s41598-022-12589-4 PMC9122962 35595795

[34] Kafi‑Ahmadi, L., Khademinia, S. Poursattar Marjani, A. & Nozad, E. Microwave‑assisted preparation of polysubstituted imidazoles using Zingiber extract synthesized green Cr_2_O_3_ nanoparticles. *Sci. Rep*. **12**, 19942 (2022). 10.1038/s41598-022-24364-6 PMC9675835 36402805

[35] Safari J, Zarnegar Z, Heydarian M (2012). Magnetic Fe_3_O_4_ nanoparticles as efficient and reusable catalyst for the green synthesis of 2-amino-4*H*-chromene in aqueous media. Bull. Chem. Soc. Jpn..

[36] Poursattar Marjani A, Ebrahimi Saatluo B, Nouri F (2018). An efficient synthesis of 4*H*-chromene derivatives by a one-pot, three-component reaction. Iran J. Chem. Chem. Eng..

[37] Ovchinnikova A, Andin AN (2013). Recyclization of dimedone adduct with 2-(2-oxo-2-phenylethylidene)propanedinitrile in the reaction with *N*-nucleophiles. Russ. J. Org. Chem..

[38] Hasaninejad A, Golzar N, Beyrati M, Zare A, Doroodmand MM (2013). Silica-bonded 5-n-propyl-octahydro-pyrimido[1,2-*a*]azepinium chloride (SB-DBU)Cl as a highly efficient, heterogeneous and recyclable silica-supported ionic liquid catalyst for the synthesis of benzo[*b*]pyran, bis(benzo[*b*]pyran) and spiro-pyran derivatives. J. Mol. Catal. A: Chem..

[39] Khoshneviszadeh M, Najmeh E, Miri R, Foroumadi A, Hemmateenejad B (2012). QSAR study of 4-aryl-4*H*-chromenes as a new series of apoptosis inducers using different chemometric tool. Chem. Biol. Drug Des..

[40] Khan TA, Lal M, Ali S, Khan MM (2011). One-pot three-component reaction for the synthesis of pyran annulated heterocyclic compounds using DMAP as a catalyst. Tetrahedron Lett..

[41] Hasaninejad, A., Shekouhy, M., Golzar, N., Zare, A. & Doroodmand, M. M. Silica bonded n-propyl-4-aza-1-azoniabicyclo[2.2.2]octane chloride (SB-DABCO): A highly efficient, reusable and new heterogeneous catalyst for the synthesis of 4*H*-benzo[*b*]pyran derivatives. *Appl. Catal. A: Gen.* **402**, 11–22 (2011).

[42] Kargar Behbahani, F. & Sadeghi, M. On water CuSO_4_.5H_2_O-catalyzed synthesis of 2-amino-4*H*-chromenes. *J. Korean Chem. Soc.* **57**, 357–360 (2013).

[43] Moradi Gorji, F. & Monadi, N. Synthesis and characterization of Cu(II) Schiff base complex immobilized on graphene oxide/α-Fe_2_O_3_ as heterogeneous catalyst for the three-component synthesis of 2-amino-4*H*-chromenes derivatives and dye reduction. *Synth. Met.* **258**, 0379–6779 (2019).

