# Supplementary material for: Fe3O4@Glycerol-Cu as a novel heterogeneous magnetic nanocatalyst for the green synthesis of 2-amino-4H-chromenes
Source: Sci Rep. 2022 Dec 22;12:22173. doi: 10.1038/s41598-022-26769-9 (PMC9780244; doi:10.1038/s41598-022-26769-9)
Supplement: Supplementary file 1 — Supplementary Information. [file 41598_2022_26769_MOESM1_ESM.pdf]

Supporting Information

Fe_3O_4@Glycerol-Cu as a novel heterogeneous magnetic nanocatalyst for the green synthesis of 2-amino-4*H*-chromenes

Ahmad Poursattar Marjani*, Fatemeh Asadzadeh and Aria Danandeh Asl

Department of Organic Chemistry, Faculty of Chemistry, Urmia University, Urmia, Iran

*E-mail: a.poursattar@urmia.ac.ir; a.poursattar@gmail.com

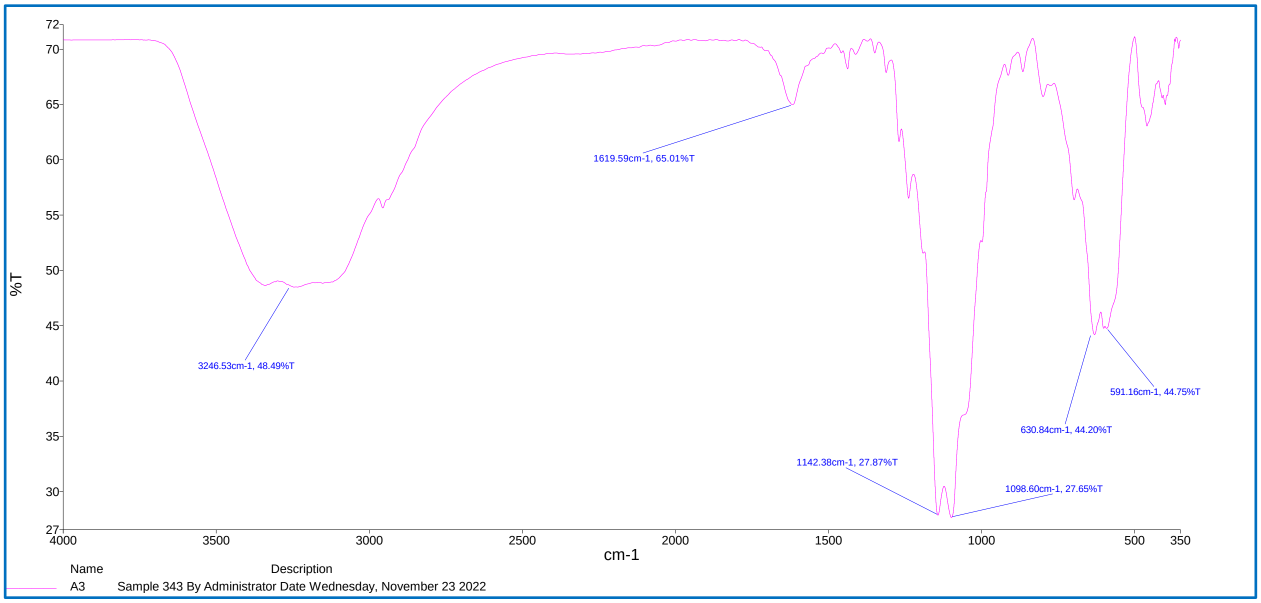

FT-IR spectrum of the 6th recovered Fe_3O_4@Glycerol-Cu

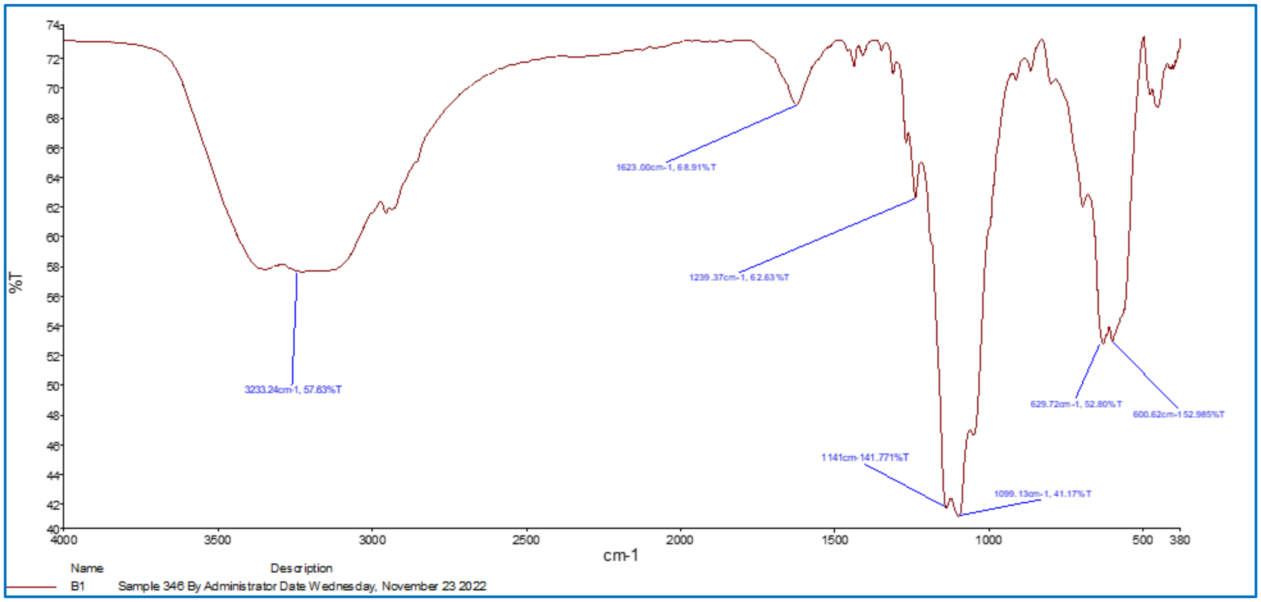

FT-IR spectrum of the 1st recovered Fe_3O_4@Glycerol-Cu

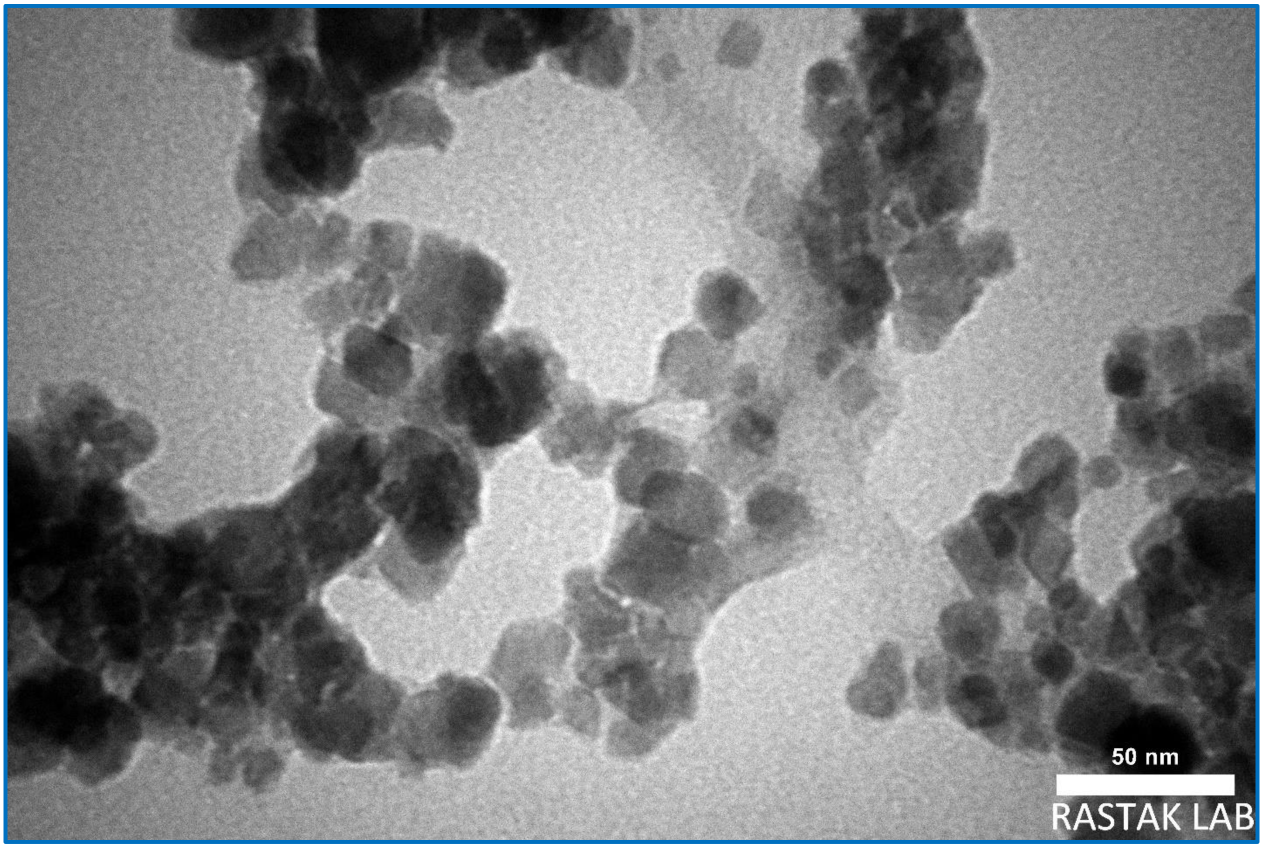

TEM image of recycled Fe_3O_4@Glycerol-Cu

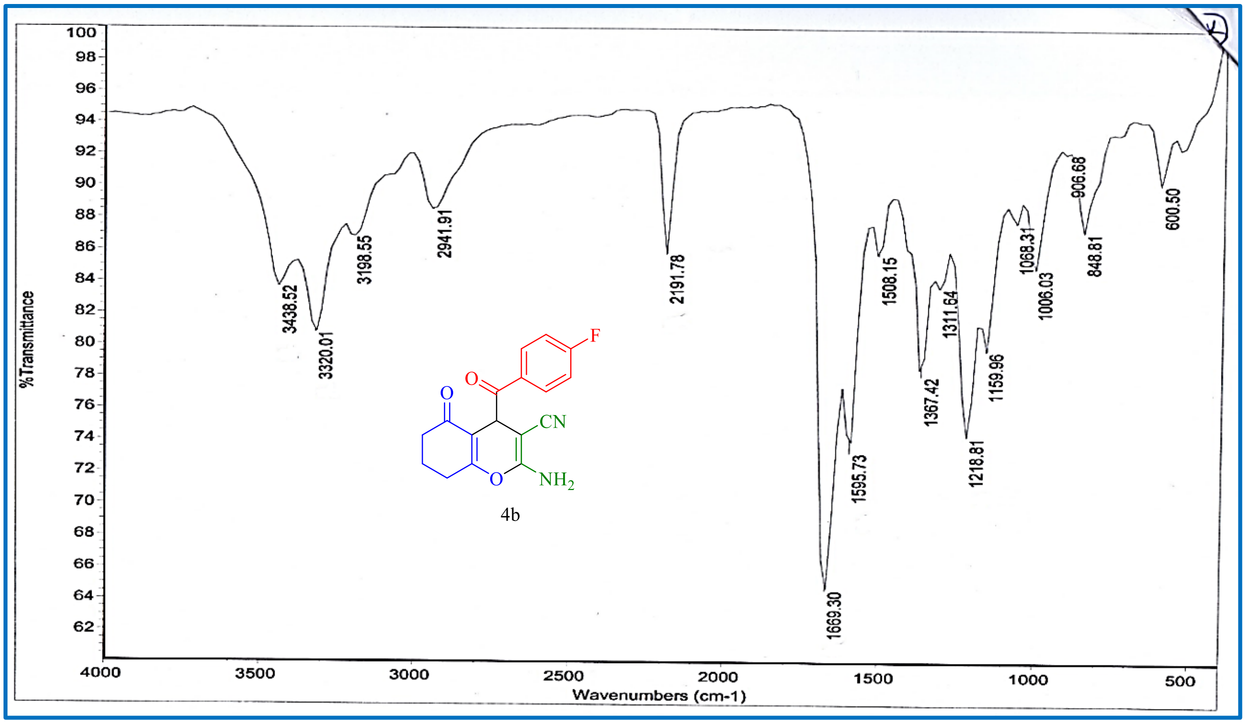

FT-IR spectrum of compound **4b**

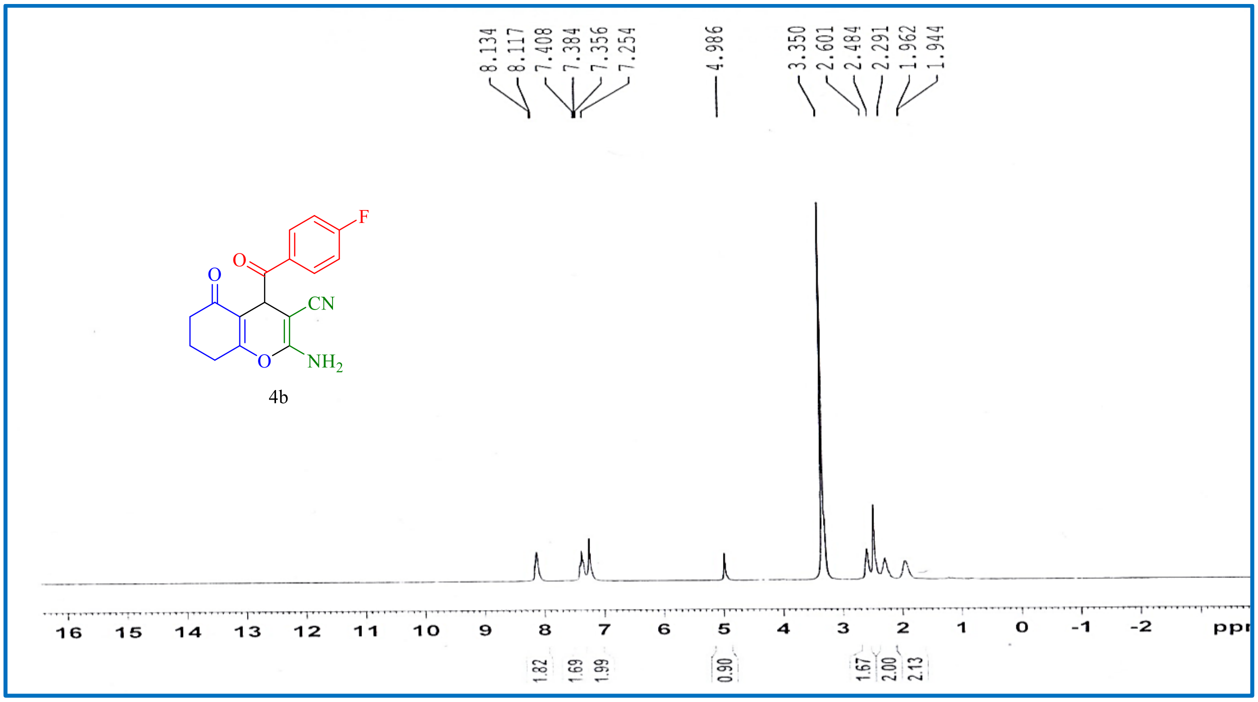

^{1}H-NMR spectrum of compound **4b**
